# Single center retrospective clinical audit and comparison of outcome between epicardial and transvenous endocardial permanent pacemaker implantations in dogs

**DOI:** 10.1371/journal.pone.0290029

**Published:** 2023-11-28

**Authors:** Liza S. Köster, Xiaojuan Zhu, Christopher K. Smith, Josep Aisa

**Affiliations:** 1 Small Animal Clinical Sciences, University of Tennessee College of Veterinary Medicine, Knoxville, Tennessee, United States of America; 2 Office of Information Technology, University of Tennessee, Knoxville, TN, United States of America; University Medical Center Groningen, University of Groningen, NETHERLANDS

## Abstract

The aim of this retrospective cohort study was to provide a single-center clinical audit of complications for single chamber permanent pacemaker implantation (PPI) techniques and determine if the clinical parameters, PPI technique or complications were associated with outcome. The electronic medical records were searched for dogs treated for bradyarrhythmia with PPI. Data related to presenting complaint, signalment of the dog, ECG diagnosis, echocardiographic findings, PPI technique, and programing of the pacemaker were recorded. Survival length (days) was recorded as the last veterinary visit; if the dog was dead the reason was documented. Cumulative survival of each pacemaker was examined by a Kaplan-Meier survival curve and the two techniques compared with a logrank test. Chi-square was used to determine the association between major complications and death. A total of 66 dogs with 52 transvenous and 30 epicardial PPIs were included. All epicardial pacemakers were implanted via transdiaphragmatic approach. A total of 31 life-threatening complications were reported. There were nine deaths related to major complications (13.6% of the study sample). The median follow-up period was 366 days, with a median survival of 255 days, and a significant difference in cumulative survival of each pacemaker (P = 0.01) between epicardial (93 days, range 0–1882 days) and transvenous (334 days, range 0–2745) PPIs but no significant difference in cumulative survival between the two techniques when only the first pacemaker was considered (p = 0.07). The presence of a major complications had a significant association with death due to pacemaker complications (P<0.001). The decision to perform epicardial PPI in failed transvenous PPI patients may have skewed the cumulative survival as was evident in the lack of significant difference in survival when only first PPI were examined. Major complication rates between the two techniques were similar and the authors consider both techniques equally reliable to manage symptomatic bradycardia in dogs.

## Introduction

Permanent pacemaker implantation (PPI) in dogs is indicated for the management of symptomatic bradyarrhythmia, including high grade second-degree atrioventricular (AV) block, complete AV block (CAVB), both paroxysmal and persistent forms, and sick sinus syndrome (SSS) that no longer responds to medical management [1]. Less commonly, the need arises in rare conditions including persistent atrial standstill and in reflex syncope [1–7]. Techniques described include transvenous endocardial and epicardial placement [8–14]. When the transvenous technique is contraindicated due to reasons that include, but not exclusive to, the size of the patient, hypercoagulability, failure of venous access, diagnosis of vegetative endocarditis, concurrent use of immunosuppressive drugs, and dermatological or other infection over the venous access site, then epicardial implantation is preferred [9, 15].

Outcome and complications of cardiac pacing in dogs using single chamber pacing, including both epicardial and endocardial techniques of PPI, with programming as ventricular demand pacing, have been published in the veterinary literature. The largest outcome study is a multicenter retrospective published in 2001, where major life-threatening events related to loss of pacemaker function were documented in 33% of dogs [16]. In that study, the percentage contribution of epicardial PPI was low, 12%, and the contribution of each technique to outcome was not reported. The subjective impression of the authors of an earlier study published in 1991 was that transvenous pacemaker implantation was feasible and a less traumatic alternative to epicardial pacemaker implantation [17]. A direct comparison of the two techniques was not available, instead, the authors supported their arguments by comparing their outcome to on previously reported complications for epicardial pacemaker implantation [13, 18, 19]. Strong opinion considers transvenous PPI as the preferred technique in the canine patient due to ease of placement and high success rate [7, 16, 20, 21]. Despite this recommendation, outcome studies of transvenous PPI in dogs report major complication rates varying from 14–35%, and one report of periprocedural death of 6.4% [7, 17, 20, 22–24]. Reports with epicardial PPI cases often describe different surgical techniques in few patients, making it difficult to draw collective conclusions. Major complication rates have been reported in 25 to 72% of patients, with periprocedural death of 28% in one report [8, 9, 15, 18, 25, 26]. The epicardial transdiaphragmatic surgical approach is considered rapid, cosmetic and subjectively less painful compared to other epicardial PPI techniques [18]. Despite a major complication rate (25%) and survival time that was similar to those reported for transvenous pacemaker implantation (13–33% reported at the time of their publication [7, 15, 16, 20]), and an increased major complication rate in dogs > 10 kg related to lead dislodgement, a study concluded that this technique was an acceptable alternative option to transvenous PPI placement [15].

A direct comparison of risks and complications between transvenous and a single standardized epicardial technique in a single center has not been previously reported. Our veterinary teaching hospital historically has had a unique cohort of dogs that underwent epicardial PPI via transdiaphragmatic surgical technique, not necessarily due to contra-indications related to a transvenous PPI method, but because of historical limitations of the available personnel to perform a transvenous procedure. Therefore, in this is single institution, complication rates between the two groups could be directly compared. The primary aim of this retrospective study was to describe anesthetic time, temporary pacing time, anesthetic complications, surgical and post-operative complications, and pacemaker programing/sensing complications for single-chamber, VVI/VVIR, transvenous and epicardial pacemakers. The second outcome was to examine the effect and the cumulative survival of pacing techniques. In addition, the associations between selected clinical parameters, and complications with pacemaker related death were examined. The authors of this paper hypothesized there would be no differences in the complications and cumulative survival between transvenous and epicardial permanent pacemaker implantation for the management of symptomatic bradycardia.

## Materials and methods

This was a single institution retrospective cohort study using the electronic health record database of the University of Tennessee from April 2012 to March 2022. Animal owners sign a hospital consent form upon checking in that data from the medical record and imaging studies pertaining to their pet can be used for research and publication. An excerpt from the current consent statement reads as follows, ’I hereby authorize the University of Tennessee College of Veterinary Medicine and its authorized designees to record, transmit, and store in any format and/or medium (analog, digital, electronic, or other media now known or later developed) my own and my animal’s name, likeness, images, voice, appearance, data, diagnostics, and/or medical records arising out of and relating to the performance, care, and medical treatment hereunder and, further, hereby grant the institution and its authorized designees the perpetual and irrevocable right to use the same, in whole or part, for any commercial or non-commercial purpose including, but not limited to, research’. The electronic medical record database was searched for canine patients with a diagnosis code of ‘permanent pacemaker implantation’. Exclusion criteria included incomplete medical record or indications for PPI other than symptomatic bradycardia.

Patient information collected included signalment, presenting complaint, ECG diagnosis, concurrent structural heart disease, anesthetic and temporary pacing time, PPI technique, lead type, fixation technique, suture size (diameter) for fixation of the lead tip to the epicardium or the collar to the jugular vein for epicardial and transvenous technique respectively, pacing mode and rate, and use of antibiotics. Demographic variables recorded included age in months, weight in kg, sex and neuter status, and breed. The electrocardiographic diagnosis of symptomatic bradycardia was categorized as one of the following diagnoses: CAVB, high-grade second-degree AV block, infranodal 2:1 AV block, SSS, or atrial standstill. A 2:1 AV block was considered infranodal if not exercise or vagolytic responsive, the patient had clinical signs, or a 24 hr ECG demonstrated progression to a more advanced AV-block. If an atropine response test was documented in the electronic medical record it was recorded as was the interpretation as responsive (post-atropine heart rate is ≥ 150 or at least a 50% increase in heart rate from baseline) or not [27]. Other information collected included the presence of a pre-existing cardiac disease documented in the patient examination and echocardiogram, whether the procedure was considered an emergency or elective, and the implantation technique as either epicardial or transvenous endocardial. If the transvenous technique was employed, whether an active or passive lead fixation technique was selected. Total anesthetic time until PPI was completed, and temporary pacing time were calculated from the anesthetic monitoring sheets. Complications were categorized as either anesthetic, surgical related and further sub-categorized as major or minor, depending on if they were considered life-threatening or not. In the case of anesthesia complications, hypotension was defined as a systolic of < 90 mmHg. The temporary pacemaker technique i.e. transvenous versus transthoracic, and associated complications was recorded. Post PPI examinations with the cardiology service were examined and data related to generator interrogation, and complications were recorded if present, as programming related, acute loss of capture either due to lead dislodgement, or failure to capture due to rising threshold, malfunction or fracture of the lead, lead thrombus, ventricle perforation, or device associated infection. Failure to capture due to rising threshold was confirmed by a pacing spike with absent myocardial depolarization, either at the time of implantation or evidence of a chronic rise in threshold demonstrated by interrogation. A dog could potentially be allocated several major complications. These were then classified as major, if they were considered life-threatening, or necessitated replacement of the PPI, or minor, if not.

Attempts to determine follow-up and survival time (in days) were made by examining the last entry in the medical record. If the last visit for pacemaker interrogation was within the scheduled time frame, this was recorded. If the patient status was not known, due to the time lapsed from the last visit exceeding the anticipated revisit schedule, or in the scenario where a dog would be examined by a cardiologist or pacemaker technician for interrogation at a site other than our hospital, the referring veterinarian was contacted to determine the status of the animal. If the animal was deceased, the reason and date of death were recorded, as well as if the death was pacemaker complication related or not.

### Statistical methods

Descriptive statistics were used for all variables with the median and range reported (JMP^®^, version 16.0.0, SAS Institute Inc., Cary, NC). Data was separated into two age categories (young dogs under 72 months of age versus older dogs) guided by the findings by Wess *et al*. (2006) and two weight categories (at least 10 kg and greater than 10 kg) guided by the findings by Visser *et al*. (2013). A Chi-square test was performed to determine the association between either the age group or body weight group, and major complications; and between the PPI technique and the clinical signs (JMP^®^, version 16.0.0). Complications related to temporary pacing technique i.e., transvenous versus transthoracic, were compared using the Mann-Whitney U test (JMP, version 16.0.0). The association between major complications and the implantation lead type, as well as the suture material thickness and lead dislodgement, was tested using Fisher’s exact test (JMP, version 16.0.0).

Survival analysis was performed by Kaplan-Meier survival curve and logrank test to compare the differences in the survival time between the type of pacemaker implantation i.e., epicardial versus transvenous (SAS, 9.4, TS1M7, SAS Institute Inc., Cary, NC). The survival analysis was repeated when only the first pacemaker was included, to exclude the bias of death related to a scenario of acute loss of capture, without lead dislodgement, that persisted despite repeated implantation, which is independent of the technique. The logrank test was used to test the effect of age, sex, structural heart disease, and complication by each type of pacemaker, type of clinical signs and the number of clinical signs (SAS, 9.4, TS1M7). Deaths related to pacemaker failure was considered as the event. All other causes of death were considered censored. P <0.05 was considered significant.

## Results

### Presentation and signalment

Sixty-nine dogs underwent a PPI during a 10-year period at a single academic institution. Three were excluded due to lack of clear indications for PPI or incomplete medical records. Of the 66 remaining dogs, 17 (25.75%) were mixed breed dogs, 10 (15.15%) miniature schnauzers, 5 (7.58%) Labrador retrievers, 4 (6.06%) Boston terriers, 3 (4.55%) of each of the boxer, cocker spaniel, German shepherd, and jack Russel terrier breed, 2 (3.03%) of each of the dachshund, bulldog, golden retriever, and Maltese breed, and 1 (1.52%) of each of the Bichon Frise, cairn terrier, Doberman, Dogue de Bordeaux, German shorthair pointer, great Dane, Irish terrier, pug, Shetland sheepdog, and Weimaraner breed.

The median age of the population was 125 months (range 36–180). The population was comprised of 42 females, of which 41 (97.62%) were spayed, and 24 male dogs, with 22 (91.67%) neutered. Client complaint and reason for referral were recorded as total loss of consciousness (TLoC) or collapse (n = 42, 63.64%), exercise intolerance and/or lethargy (n = 20, 30.30%), gastro-intestinal signs (n = 4, 6.06%), and/or miscellaneous (n = 6, 9.09%). In 6 dogs (9.09%), the bradycardia was an incidental finding on clinical examination by the referring veterinarian, and subsequently diagnosed with advanced AV blocks with a potential risk of sudden cardiac death, warranting artificial pacing. In 25 dogs (37.88%), the PPI was considered an emergency procedure, while 41 dogs (62.12%) were presented for a routine appointment for PPI.

Table 1 depicts the median, minimum, maximum and range heart rate (bpm) for each category of symptomatic bradycardia and the type of pacemaker technique initially selected to correct it.

**Table 1:** Etiologies and heart rates of dogs that underwent either epicardial or transvenous permanent pacemaker implantation.

Indications for pacing	PPI technique	N	Median (bpm)	Range
CAVB	Epicardial	13 (20%)	37.5	40
CAVB	Transvenous	25 (38%)	44	98
2ndAVB	Epicardial	6 (9%)	55	70
2ndAVB	Transvenous	8 (12%)	45	42
2:1	Epicardial	0 (0%)	NA	NA
2:1	Transvenous	2 (3%)	100	0
SSS	Epicardial	3 (4%)	80	12
SSS	Transvenous	9 (14%)	70	70

The indication for permanent pacemaker implantation (PPI) and the corresponding heart rates (bpm) and ranges associated with each etiology of bradycardia. The data is divided by the initial surgical approach, epicardial vs. transvenous. The number of animals, expressed as a percentage of the total in parenthesis, is listed according to the bradyarrhythmia diagnosis and listed according to the category of PPI technique.

PPI, permanent pacemaker implantation; bpm, beats per minute; Min, minimum; Max, maximum; CAVB, complete atrioventricular (AV) block; 2ndAVB, second degree AV block; 2:1, two to one AV block; SSS, sick sinus syndrome.

There was no significant association between the clinical signs and the type of pacemaker technique chosen by the clinician (P = 0.06). According to the log-rank test, there was no difference between the clinical signs in the survival probability due to pacemaker implantation at any time point (P = 0.14), and there was no significant effect of the number of clinical signs in the survival probability due to pacemaker implantation at any time point (P = 0.20).

### ECG and echocardiographic diagnosis

**Fig 1:**
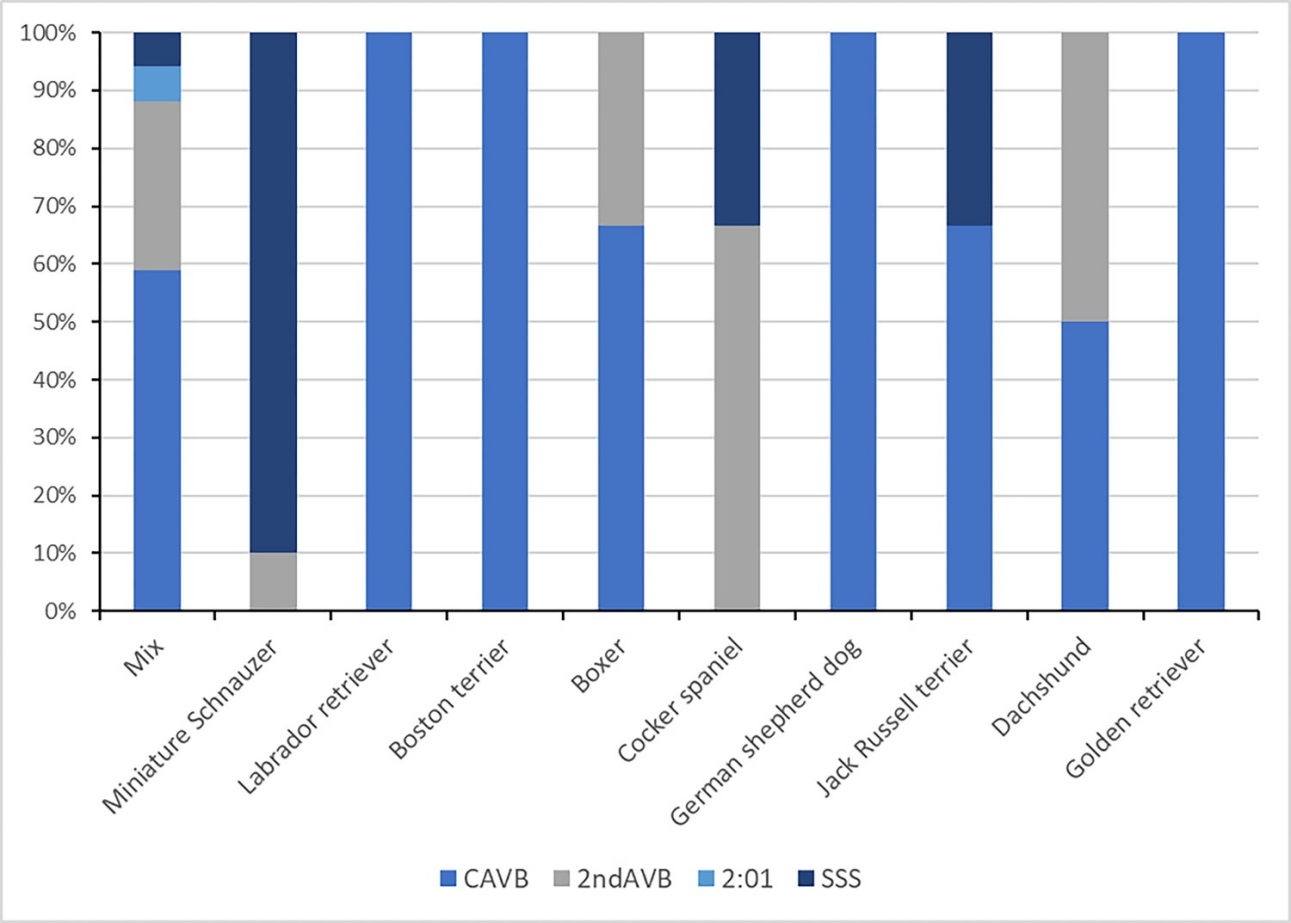
Association of the 10 most common dog breed and etiology of bradycardia. Legend: The ten most common breeds, with mixed breed considered a breed for the purposes of this study, are included. The shading corresponds to a category of bradycardia as per the ECG diagnosis. CAVB, complete atrioventricular (AV) block; 2^nd^ AV block, second degree AV block; 2:1, 2 to 1 AV block; SSS, sick sinus syndrome.

The association between the 10 most common breeds and their ECG diagnoses is depicted in Fig 1. Twenty-five dogs had a documented atropine response test performed either by the referring veterinarian or at our institution: Only 7 (28.00%) dogs responded; 2 dogs (16.00%) were diagnosed with CAVB, 2 dogs (25.00%) were diagnosed with second-degree AV blocks, and 3 (60.00%) dogs had SSS. The post-atropine ECG was not available for review and the response could not be verified but was documented by the clinician in the medical record.

Concurrent heart disease or abnormalities described on the echocardiography report at the time of diagnosis of bradyarrhythmia, are listed. Fourteen dogs (21.21%) had cardiomegaly documented in the initial echocardiogram as either left ventricular dilation and left atrial enlargement, or subjective appearance of all chambers being enlarged in the absence of valvular disease or systolic dysfunction. The cause was likely to be related to the bradycardia rather than primary structural heart disease, as the normalized dimensions of the left ventricle and atrium were within reference intervals on follow-up examination post-PPI in many of the dogs and the absence of valvular disease or subnormal systolic function. Two additional dogs (3.03%) had isolated tricuspid insufficiency with normal valve morphology, and ascites, consistent with right-sided congestive heart failure (R-CHF), and related to the bradycardia, as the signs of R-CHF resolved after PPI in one dog and the second dog had an escape rate of 25 bpm and suffered a peri-procedural death. A total of 29 dogs (43.94%) had a description of structural heart disease on echocardiogram. Volume overload could have caused misclassification of disease stage and is therefore not stated. Twenty-three dogs (34.85%) had myxomatous mitral valve disease (MMVD), 2 dogs (3.03%) had dilated cardiomyopathy (DCM) phenotype, 1 dog (1.52%) had a tentative diagnosis of a cardiac associated tumor (chemodectoma), 2 dogs (3.03%) had congenital disease (tricuspid dysplasia and pulmonic valvular stenosis), and 1 dog had pulmonary hypertension (1.52%).

Additional recorded ECG abnormalities included ventricular ectopy (n = 7, 10.61%), concurrent to either CAVB or high-grade second-degree AV block. Three of these dogs (42.86%) had ventricular tachycardia, 2 (28.57%) had advanced MMVD and 1 dog (14.29%) had R-CHF without structural heart disease diagnosed. Four dogs (57.14%) had singlet ventricular premature complexes (VPCs), with a boxer with VPCs having a structurally normal heart but later presumptively diagnosed with arrhythmogenic ventricular cardiomyopathy; and the other 3 dogs (42.86%) having no structural heart disease.

### Implantation technique, anesthetic time, and pacemaker settings

Sixty-six dogs had a total of 82 pacemakers implanted, 30 (36.59%) epicardial and 52 (63.41%) percutaneous transvenous endocardial. In the transvenous endocardial technique, all leads were single chamber; a total of 26 (50.00%) tined and 19 (36.53%) active leads were used, with the remainder not documented in the surgical report. Medtronic Inc. (Minneapolis, MN) was the exclusive manufacturer for all the pacemaker leads and generators. All leads (CapsureFix MRI SureScan, Medtronic, Minnesota, USA) and generators (ADVISA ADAPTA, SENSIA, MRI SureScan Pacing System, Medtronic, Minnesota, USA) were first time use and sourced from Companion Animal Pacemaker Registry and Repository.

For the epicardial PPI, a transdiaphragmatic surgical approach was performed in all cases as previously described by Visser *et al*. [15]. Stay sutures were routinely placed on each edge of the pericardiotomy incision to facilitate caudal translation of the heart and placement of the epicardial sutures. Polypropylene (Prolene®, polypropylene, Ethicon, Cincinnati, OH) suture material was used to secure the lead button to the left ventricle in all cases. Suture size was 3/0 or 4/0 USP, at the discretion of the surgeon. The lead was attached to the generator and secured in a muscular pocket in the left body wall as described previously [15]. For transvenous endocardial technique, where the generator was secured in a lateral cervical subcutaneous pocket as described by Estrada *et al*. (2019) [10], the left jugular was used to introduce the lead in 34 dogs (51.52%, 37 pacemakers), and the right jugular in 14 dogs (21.21%, 15 pacemakers). Four dogs (6.06%) had the jugular lead replaced in a subsequent surgery, two dogs (3.03%) on the same left side, one on same right side, and one replaced from right to left side. Silk (Perma-hand® silk, Ethicon, Cincinnati, OH) suture material was used to secure the lead to the jugular vein in 32 dogs (61.15%) and polypropylene in 14 dogs (26.92%). In four dogs, the type of suture material was not reported. Four transvenous leads dislodged using silk and four using polypropylene. Poliglecaprone 25 (Monocryl, undyed monofilament, Ethicon, Cincinnati, OH) was used to close the subcutaneous pockets created for the generator in the case of the transvenous PPIs and poliglecaprone 25 or polydioxanone (PDS-II, Ethicon, Cincinnati, OH) was used to close the abdominal wall pocket for the transdiaphragmatic epicardial technique.

A total of 82 anesthesia records were examined for 82 PPIs placed on 66 dogs. A total of 10 dogs had two or more anesthetics (one dog required 4, four dogs required 3, and five dogs required 2 anesthetic events due to major complications) for PPI exchange; with 32% of major complications in this study requiring PPI revision in 15% of the study group. One dog had four pacemakers implanted in 24 hours, with two separate attempts at transvenous PPI implantation, followed by two epicardial PPI placements, and all failures due to dislodgement. Four dogs had three pacemakers placed over a period spanning a few hours to several weeks. The reasons included repeated failure to capture in the presence of pacing spikes and presumed high threshold in two of the dogs, and repeated transvenous dislodgement in the other two dogs. Five dogs required PPI placement twice. Eight of the 10 dogs that required 2 or more anesthetic events, including the five requiring 2 anesthesias, PPI replacement was necessary due to acute loss of capture on recovery or in the short-term post-operative period (< 30 days), caused by lead dislodgement or malposition, exit block or lead failure. The remaining two dogs had replacement of the PPI for suspected endocarditis in one dog, later confirmed as a lead thrombus with pulmonary embolism on necropsy; and generator and lead malfunction that led to syncopal events due to SSS in the other dog.

Median anesthetic time for the study sample was 125 min (range 60–300 min), with median anesthetic time for epicardial PPI of 122.5 min (range 60–200 min) and transvenous PPI taking 125 (range 70–300 min) minutes. Transthoracic versus transvenous temporary pacing was used at the discretion of both the attending anesthesiologist and cardiologist. Patient size (i.e., more current is required to capture the heart rhythm as the patient gets larger), was considered when choosing one technique or the other but ultimately not the deciding factor. Cisatracurium (Nmbex, GSK plc, London, UK) was used as adjunct muscle relaxation at the attending anesthesiologists’ discretion. The median duration of temporary pacing for all PPI procedures was 75 min (range 0–150 min), epicardial 65 min (0–150 min) versus transvenous 85 min (range 15–150 min). Temporary pacing was transthoracic in 33 and transvenous in 37 anesthetic events. Transvenous pacing was performed via the jugular vein in 7 dogs, or femoral vein in 23 dogs, and in 7 dogs it was not recorded on the anesthetic sheets.

Pacemakers were programmed according to the preference of the attending cardiologist; demand pacing was selected in all cases, with VVI in 61 dogs (92.42%) and VVIR in 5 dogs (7.58%), and with initial rates of 60 bpm (n = 2), 70 bpm (n = 8), 80 bpm (n = 21) and 100 bpm (n = 31). At 1-month re-examination, VVI pacemakers were re-programmed to VVIR and all dogs had VVIR with rates (bpm) ranging from 60 to 100.

### Complications

A total of 31 (37.80%) major or life-threatening complications and 15 (18.20%) minor complications were recorded in 82 PPIs. Complications were listed according to the following categories: pacemaker generator and lead, and surgical complications. The numbers are divided according to technique (epicardial versus transvenous endocardial) and detailed in Table 2. There were 26 (26/31, 83.87%) major or life-threatening complications in 15 dogs related to the pacemaker generator or lead of the total 82 PPIs, and 5 (5/31, 16.12%) major complications related to surgery. For minor complications, 10 (10/15, 66.66%) were related to the generator or lead, and five related to surgery (5/15, 33.33%). Major complications related to PPI were not influenced by the age of the dogs (P = 0.30). A significant association between body weight and life-threatening complications (P = 0.015) was found. Dogs weighing more than 10 kg body were more likely to have life-threatening complications (25 times/131 visits, 19.00%) than dogs less than or equal than 10 kg (6/84 visits, 7.14%).

**Table 2:** Categories of pacemaker complications.

Category of complication	Pacemaker and lead		Surgical	
	Major	Minor	Major	Minor
**Epicardial**	11 (11/30, 37%)	2 (2/30, 7%)	2 (2/30, 7%)	2 (2/30, 7%)
**Transvenous**	15 (15/52, 29%)	8 (8/52,15%)	3 (3/52, 6%)	3 (3/52, 6%)
**Total**	26	10	5	5

Categories of pacemaker complications in 66 dogs and 82 PPI, including the following groups: pacemaker lead and generator, anesthesia and temporary pacing, and surgical related complications. In parenthesis is the percentage of complications per subcategory for both epicardial (n = 30) and transvenous (n = 52) techniques.

The most common generator and lead related major complication was acute loss of capture, due to lead dislodgement (n = 11) and those not related to lead dislodgment (n = 11) either due to lead malfunction or exit block (Table 3). Lead dislodgement was diagnosed by lack of pacing spikes, and/or radiographic evidence, either on thoracic radiographs or screening fluoroscopy. Eight events of lead dislodgement were transvenous and three epicardial, with the median time interval from implantation until lead dislodgement of 1-day (range of 0–46 days) for transvenous and 15-days for epicardial (range of 0–30 days). Five transvenous endocardial lead dislodgements were tined versus one which was active; 2 were not recorded. There was no significant association between the lead type and occurrence of a major complications, with 15.00% of the active leads (3/20) and 15.38% of the tined leads (4/22) having major life-threatening complications (P = 1.00). There was no association between suture size and the dislodgement of epicardial (P = 0.60) or transvenous (P = 1.0) PPIs. One transvenous and one epicardial lead dislodgement were associated with ventricular perforation. The transvenous perforation was an active lead. Acute loss of capture without dislodgement occurred as 11 events with four transvenous and seven epicardial, with a median time from the time of implant recorded as 0 days (range 0–870 days). Nine dogs had a single lead or generator related event, six due to acute loss of capture secondary to rising threshold, one due to generator failure, one caused by epicardial ventricular perforation and one secondary to dog bite. The atypical short-term trauma-related complication due to a dog attack bite over the neck occurred one month after placement, causing the lead to become dislodged with subsequent acute loss of capture. The six dogs that had confirmed or suspected acute loss of capture due to rising threshold and included a dog that had 3 PPIs; two dogs that each required 2 PPIs, one of which was euthanized for this reason and the other one was lost to follow-up; and three dogs with 1 PPI each, one of which was euthanized.

**Table 3:** Incidence of complications related to the pacemaker lead and generator.

Complication	Number of events		
	Epicardial	Transvenous	Total
**Lead dislodgement** **Intraoperative** **Post-operative**	4 1 3	10 2 8	14
**Loss of capture without lead dislodgement** **Exit block** **Lead fracture**	7 2 1	4 0 0	11
**Generator malfunction**	1	1	2
**Ventricular perforation**	1	1	2
**Pocket infection**	0	1	1
**Lead thrombus complication**	0	3	3
**Synchronous diaphragmatic contraction**	0	2	2
**Muscle twitch**	1	0	2
**Programming error (oversensing)**	1	5	6

The number of lead dislodgements includes both intraoperative and post-operative incidences. Due to the nature of the lead dislodgement, each event could cause additional major complications including ventricular perforation and synchronous diaphragmatic contractions. Loss of capture without dislodgement could have been related to lead fracture, or rising threshold due to exit block.

Two of the dogs with lead dislodgement from transvenous placement developed synchronous diaphragmatic contraction. One suffered a right ventricular perforation and on the other the lead was pulled into the right atrium. At interrogation, the one generator with the lead dislodged into the right atrium had increased in output voltage, draining the battery life. This dog was not pacemaker dependent and was transiently managed for a 2:1 AV block by changing the generator mode to OVO to prevent discharge and muscle twitching. Epicardial pacemaker implantation was scheduled in office hours and high threshold for capture was recorded at implantation. This same dog suffered sudden cardiac death 40-days later.

Complications that occurred or were documented after the first re-examination, which is usually scheduled approximately 1-month post-PPI, were considered late occurrences of pacemaker complications and were uncommon. Three dogs had late occurrence of major complications, and included, one dog seen 15-months after PPI with a history of recurrent syncope, and a suspected generator pocket infection, although bacterial culture negative, and suspected hyperadrenocorticism. This dog had a transvenous pacemaker exchanged for an epicardial location due to the suspected generator pocket infection but continued to have syncopal events. A second dog also presented 1-year after epicardial PPI for management of syndromic SSS, due to recurrent syncopal events related to under sensing and intermittent capture, and the cause was determined to be lead or generator failure. The third dog had loss of capture, without apparent epicardial lead dislodgement 870 days after placement. Despite replacing the PPI surgically, the new lead and generator failed to capture the myocardium despite increasing the output, and the cause was a presumed exit block due to altered tissue threshold.

Minor complications included oversensing (n = 6), lead associated thrombus (n = 2), and a seroma (n = 1). Both cases of lead associated thrombi were associated with acute pancreatitis.

Anesthesia related complications included reports of hypotension in 44-, ventricular ectopy in 7-, and atrial fibrillation in 2-procedures. One dog was severely hypotensive and bradycardic and developed ventricular fibrillation which reverted to sinus rhythm after defibrillation. None of the episodes of ventricular ectopy had sustained ventricular tachycardia (greater than or equal to 3 successive ventricular ectopic beats), although ventricular tachycardia was reported as a surgical complication during epicardial suture placement. A total of 10 minor complications were recorded related to temporary pacing, and there was no significant difference between transthoracic and transvenous approach (P = 0.74). Most were related to transient failure to capture.

Surgical complications were categorized as major and minor, and according to surgical approach. Major complications, considered life-threatening, included lead dislodgment, arrythmias during suturing of the epicardial button to the myocardium, and significant hemorrhage related to vessel catheterization. Three leads dislodged intraoperatively, two transvenous and one epicardial, and are listed in Table 3. One dog suffered non-sustained ventricular tachycardia during epicardial lead suturing which, according to the anesthetic report, resolved with a splash lidocaine block. A splash block is 1-2mg/kg dose of lidocaine applied to the epicardial surface of the heart by surgeons to mitigate ventricular ectopy during direct cardiac manipulation. Severe hemorrhage was reported in one dog after a temporary lead was inadvertently advanced into the femoral artery rather than the vein and then removed causing bleeding. Minor complications were uncommon and included inability to pass the lead through the jugular vein and minor bleeding during epicardial approach.

Of the 35 deaths recorded, nine were confirmed secondary to pacemaker complications (5 epicardial and 4 transvenous), which would translate to a 25.70% mortality related directly to PPI complications, and 13.60% of the study patient sample (Table 4). One additional death was recorded by an emergency clinic where the cause of death was euthanasia due to collapse.

**Table 4:** Pacemaker associated deaths.

Pacemaker related fatal complication	Number of animals
Acute loss of capture	5
Generator malfunction	1
Ventricular perforation	1
Thromboembolism	1
Chronic rising threshold	1

The etiologies of the permanent pacemaker related deaths (n = 9), including euthanasia and sudden unexpected death, both in the immediate peri-operative period and post-operative period. Acute loss of capture was recorded if the specific etiology was not known due to lack of imaging or pacemaker interrogation before euthanasia or sudden death, and could have included lead dislodgement, lead fracture, generator or lead malfunction, and exit block.

The total PPI related deaths included three periprocedural deaths; one related to cardiopulmonary arrest after massive pulmonary thromboembolism caused by retraction of the transvenous lead during epicardial PPI placement surgery, and two euthanasia procedures performed due to acute loss of capture on anesthetic recovery despite pacing spikes. The patient that suffered cardiopulmonary arrest failed to respond to cardiopulmonary resuscitation and had been previously diagnosed with hypothyroidism. A suspected right atrial ‘vegetative lesion’ previously detected on echocardiogram was most likely a lead thrombus, dislodged by lead removal, and confirmed on necropsy. The 2 dogs euthanized due to acute loss of capture immediately following epicardial PPI were diagnosed with CAVB and had escape rates of 20 and 35 bpm respectively. One had cardiomegaly with right heart failure.

Postoperative death included one dog that suffered sudden death at home 40-days after implantation, presumably due to rising threshold and eventual failure to capture. This dog had high threshold recorded at 1-day post epicardial PPI interrogation. Post-operative euthanasia related to pacemaker complications included 3 dogs with acute loss of capture and one dog with laceration of the left ventricle caused by the epicardial lead and confirmed on CT after the dog presented to an emergency clinic with hemothorax 10-days after PPI placement. In one dog, the generator battery life ended 78-months after implantation and the owner declined replacement. Two dogs were euthanized due to the acute loss of capture despite lack of dislodgement, and had necropsies performed. One of these two dogs developed lead implantation fibrosis with trace pericardial effusion, diagnosed as a granuloma. The other dog was diagnosed with a myocardial histiocytic sarcoma, which had not been previously detected on echocardiography. These two dogs were considered as confirmed exit blocks.

### Long-term outcome

**Fig 2:**
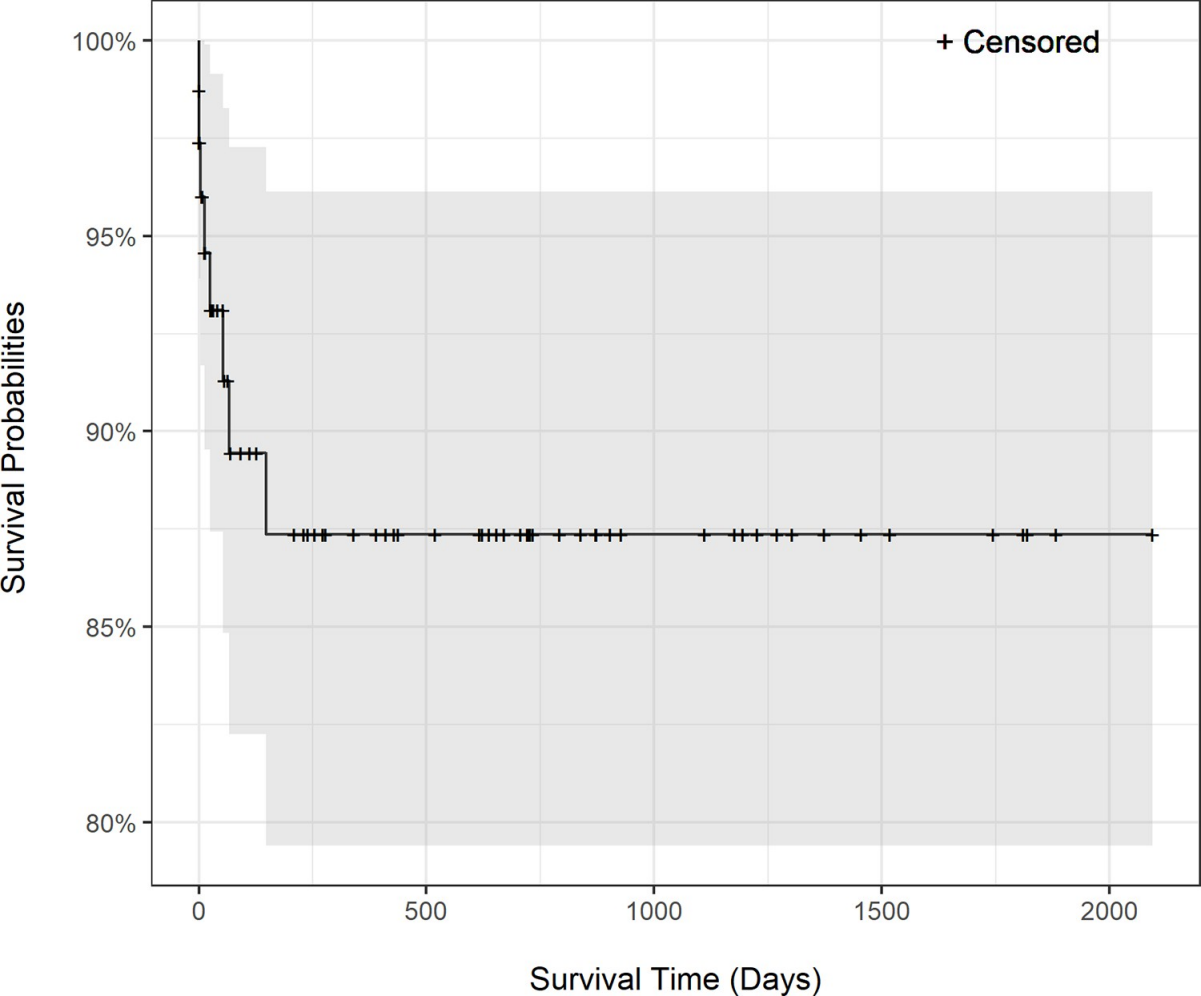
Cumulative survival. The Kaplan-Meier survival curve indicated a 60-day, 120-day, and 365-day survival probability of 91.18%, 89.30%, and 87.29% respectively, for the entire study population.

**Fig 3:**
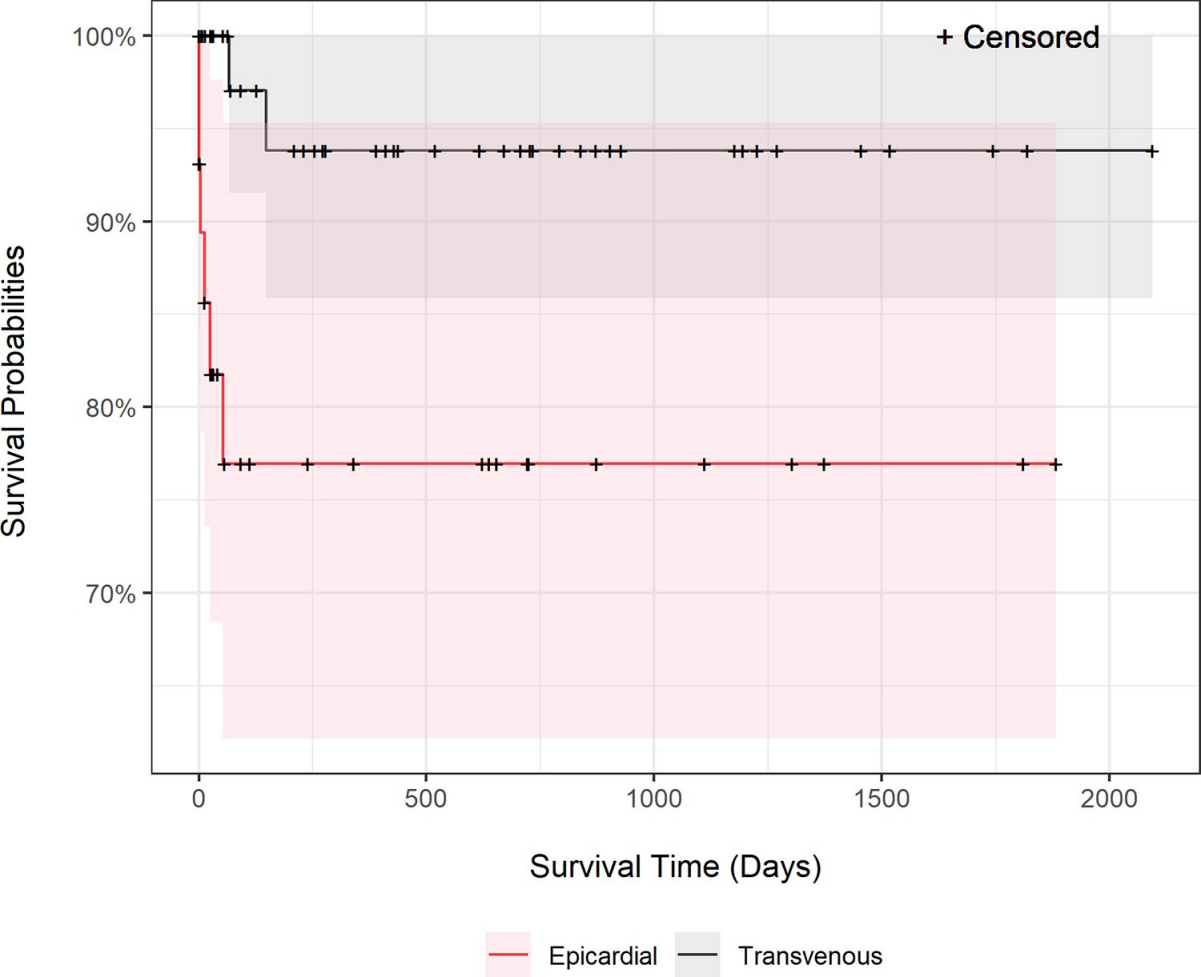
Comparison of survival probability between epicardial and transvenous PPI technique. Survival probability between epicardial versus transvenous endocardial technique was compared in the Kaplan-Meier survival curve and found to be significantly different (P = 0.01). The median survival days for the epicardial technique was 93 days (range 0–1882) and for the transvenous technique was 334 days (range 0–2745).

Follow-up data was available for 66 dogs with a median follow-up period of 366 days and a range of 0 to 2580 days. The Kaplan-Meier survival curve in Fig 2 indicated a 60-day, 120-day, and 1-year overall survival rate of 91.18%, 89.3%, and 87.3% of patients, respectively. The survival probability between epicardial versus transvenous endocardial technique was compared in the Kaplan-Meier survival curve (P = 0.01) in Fig 3. The median survival days for the epicardial technique (n = 29) was 93 days (range 0–1882) and for the transvenous technique (n = 52) was 334 days (range 0–2745). When the same analysis was performed but only considering the first pacemaker implanted, no significant difference in cumulative survival probability was found (P = 0.07) between epicardial (n = 17) and transvenous (n = 49) implantation techniques, with a median survival of 638 days (range 0–1182) for epicardial and 410 days (range 0–2745) for transvenous. The effect of age, sex, presence of concurrent heart disease (structural heart disease) on cumulative survival were examined and not found to be significant (age P = 0.41, sex P = 0.94, structure heart disease P = 0.26, indication for pacing P = 0.31). Major complications had a significant impact on survival (P<0.001 overall, P = 0.0004 for transvenous, P < .0001 for epicardial). Thirty-five dogs died due to sudden cardiac death or were euthanized due to quality of life.

In the confirmed cases of pacemaker related deaths (n = 9), the deaths occurred in < 24 hours in four dogs, in < 30 days in three dogs, and in < 180 days in one dog. One dog was euthanized due to pacemaker battery life ending at 2745 days.

## Discussion

### Indication for PPI and demographics

The results of this study in terms of population demographics and arrythmia diagnosis are consistent with previous reports, in that CAVB was the most common reason to perform PPI (38/66 dogs, 57.58%) [16, 17, 24]. Our cases differed from these previous reports in that the next most common reason for pacing was high-grade second-degree AV block in (14/66 dogs, 21.21%), with previous reports describing SSS as more common, which was only recorded in 12/66 dogs (18.18%) in our sample population. Sick sinus syndrome was represented almost exclusively by miniature schnauzers, cocker spaniel and jack Russell terrier (Fig 1), as previously reported [12, 16, 24]. As expected, CAVB presented a more mixed population with the most common breeds including mixed breed, Labrador retriever, Boston terrier, German shepherd, and Golden retriever, with the last four listed breeds exclusively presenting with CAVB [16, 20]. The Labrador retriever and the German shepherd dog have been overrepresented in previous series too [16, 20]. There was no significant association between the etiology of bradycardia and the choice of PPI technique (P = 0.0579) and choice of technique by the clinician did not appear to be influenced by clinical factors such as ECG diagnosis.

### Complications

The results of this retrospective study provide a clinical audit of complications and survival variables of 66 dogs with 82 PPI (52 transvenous and 30 epicardial) from a single institution, in a similar fashion to previous reports [7, 17, 20, 23]. One previous multi-institutional PPI study reporting complications compared outcomes between specialists and house-officers, which was not possible in our study as specialists supervised all cases and often performed parts of the procedures [16]. The chronological periods between these larger studies are not comparable either, since all report time intervals anywhere between 1984 and 2005, while our case series is more recent, from 2012 to 2022 [7, 16, 17, 20]. An exception is a previous study performed in a research setting, and spanning 2005 to 2016, for which results may not be clinically relevant [28]. A significantly shorter cumulative survival has been documented in dogs receiving reused pacemakers, which is not the case in this single center study, and could have contributed to previous reports of complications [7]. Our study timeline overlaps with only one other study (2002–2018), which examined the benefit of a surgical safety checklist on complications [22], and a report spanning 30 years (1992–2017) of PPI implantation [24]. Consideration should be given to the context of older reports, which relied on what is now considered inferior surgical techniques, reused pacemakers, less advanced lead technology, and different patient monitoring and care. Results of the last decade of PPI at our institution indicate complications remain high, despite addressing identified risk factors.

A total of 31% of the 82 PPI had major complications related to the pacemaker in 23% (n = 15) of the 66 dogs, with 6 dogs (15%) having more than a single major complication related to multiple PPIs. Complications in our study were categorized as major if they were life-threatening or necessitated lead or generator replacement, similar to two of the largest retrospective studies in dogs examining complications [7, 16]. In agreement with a previous study, dogs weighing more than 10 kg were more likely to suffer life-threatening complications (P = 0.015) [15]. Examining our data, most dogs with major complications (31% of PPI, 23% of dogs) were related to the generator or lead and required a repeat procedure. The rate of major complications in this study is comparable to Oyama *et al*. (2001) which found a 33% major complication [16], but higher than Johnson *et al*. (2006), which found a 14% of dogs suffered major complications [7]. Earlier studies examining complications related to epicardial PPI found an incidence of 40 (7/18) to 100% (11/11) [8, 18]. These latter reports likely led to the impression that transvenous is a superior technique, also related to the ease of placement, short post-operative recovery and likely more cost effective. All these studies included a small population of dogs with selection bias. Our data, which may be considered more robust, does not support this notion based on the prevalence of complications (11/30 patients, 36%), nor the lack of significant difference in lifespan from first PPI between epicardial and transvenous PPI (P = 0.67). Another study did not find a significant difference in the incidence of major complications between epicardial (28%) and transvenous (34%) implantation, although only a minority of patients had epicardial PPI (18/156, 11.5%) [16]. Our study, with a higher percentage and overall numbers of epicardial PPI (30/82, 36.5%), agrees with previous results. In our study, 36% (11/30) of epicardial PPIs had major complications, compared to a major complication rate of 28% [15/52] for transvenous PPI placement. However, at our institution, failed cases routinely had the PPI replaced with the epicardial technique skewing the data. Major complication rate for first instance PPI placement was not statistically different in this study. The implementation of a surgical safety checklist has shown to significantly reduce major complications in transvenous PPI; 15.6% versus 2% major complication rates, significantly decreasing the risk for lead dislodgement, generator infection and migration and lead perforation [22]. This surgical safety checklist was implemented by the authors at this institution in 2020, but comparison of complications before and after implementation were not examined.

The types of major pacemaker complications were similar to previous studies in that lead dislodgement and acute loss of capture due to rising threshold were the most common. Overall, 13% (11/82) of PPIs had leads dislodge causing post-operative acute loss of capture requiring the need to replace or reimplant the lead in a subsequent procedure. A previous multi-institutional study had a 10% lead dislodgement with 14 transvenous and 1 epicardial (15/154), with roughly equal number of tined and active leads dislodging [16]. Other studies had negligible lead dislodgement, with rates cited at high volume implantation centers of 3.5% (7/199) [22], 4.8% (5/104) [7] and 6% (7/105) [20]. Generator failure was cited as an important cause of pacemaker failure by Oyama *et al*. (2001) in 6% (10/154) of cases but only 1.9% (2/104) of cases by Johnson *et al*. (2007) [7, 16]. In our study, only one dog had a confirmed generator or lead malfunction due to the evidence of intermittent lack of pacing and sensing one year after implantation. Micro- lead dislodgement and lead malfunction may explain the acute loss of capture in some cases, but exit block may explain others, either due to preexisting or acquired myocardial pathology. In our study, acute loss of capture without lead dislodgement occurred in 13% (11/82) of the PPI, 6 epicardial and 4 transvenous, with three dogs (5 pacemakers) having confirmed exit block. Exit block was strongly suspected in some of these cases since replacing the pacemaker and attempting an alternative implantation technique did not correct the problem. Two exit blocks with epicardial leads were confirmed on necropsy, one associated with a myocardial histiocytic sarcoma and the other had a granuloma at the site of lead tip implantation. Exit block has been previously reported in dogs and is largely thought to be due to fibrosis surrounding the lead tip and is associated with recurrent failure to capture despite lead replacement [16, 18, 29–31]. This was confirmed in one of the two cases that went for necropsy. Steroid elution of lead tips has been utilized since 1988 to reduce this complication [32, 33]. The high number of acute loss of capture in our study was due to one dog that needed multiple PPIs and another dog with a myocardial sarcoma and, although previously reported, the latter is an uncommon condition [7].

Ventricular perforation, a major complication, was responsible for one death due to hemothorax (epicardial PPI), and one case of synchronous diaphragmatic contraction in another (transvenous PPI), in this study. Lead perforation has been previously described with transvenous endocardial active leads [16, 22, 24]. Infection was rare in our study, with only one dog diagnosed (1/66, 1.5%). Previous studies report rates of generator infections ranging from 0–15% and the use of surgical checklist may help reduce the incidence of infection by ensuring the appropriate use of perioperative antibiotic [16, 17, 22, 30, 31]. Most pacemaker infections are diagnosed between one- and four-months post implantation [17], although persistent pyrexia for 20-months post implantation with confirmed endocarditis has also been documented [7]. Despite our institution’s high major complication rate, infection was negligible and in the one unconfirmed case reported in our study was likely related to hyperadrenocorticism.

Lead fracture has been previously reported related to transvenous PPI [16], we also report a lead fracture in one epicardial PPI for the first time. Another rare complication that we report and that has been previously described is loss of capture related to neck trauma from a dog bite [20, 24]. Despite the claim that the thoracic wall rather than lateral cervical generator implantation is associated with lower rate of infections and seroma development (3% compared to 12%) [20], only one late suspected generator infection and one seroma were reported in this study, with all generators placed in the cervical location.

Described surgical approaches for epicardial pacemaker implantation in dogs include techniques that are reported to be associated to increased surgical time and trauma such as lateral thoracotomy [12, 18, 19] and caudal median sternotomy combined with cranial midline celiotomy [18], and less invasive techniques such as the trans-diaphragmatic (TD) [8, 15, 25], minimally invasive transxiphoid [26, 34], and minimal incision thoracotomy (minithoracotomy) [9] approaches. Historically, the transdiaphragmatic approach has been favored by many surgeons due to low morbidity and ease of placement and was the exclusive epicardial PPI technique used in this case series. Poor electrode implantation has however, been reported, increasing the risk for lead dislodgement [9, 15]. The leading complication (54%) reported in one study for canine epicardial PPI was cardiac arrhythmia related to the epicardial suture, with ventricular fibrillation as the etiology of perioperative death in two dogs in the same study [15]. Ventricular tachycardia controlled with a lidocaine splash block was reported as a surgical complication in one dog in this study population.

The anesthesia related complications were similar to a previous report in the literature [35]. Our study contributed to the literature in that differences in anesthesia related complications were no different between transthoracic and transvenous temporary pacing strategies (P = 0.735).

### Survival and long-term outcome

Status for all dogs was available with a median of 366-days follow-up. The median survival (255 days or 8.5 months) was shorter than previous reports for transvenous VVI (median of 14–19.9 months) [23, 36] and for epicardial (median 32.2 months) [15] techniques. The Kaplan-Meier survival curve predicted a 60-day, 120-day, and 365-day survival rate of 91.18%, 89.3%, and 87.3% respectively that was similar to previous report of a 1-year survival include 70% [16], and 86% [7]. The shorter median survival for the epicardial technique (93 days, range 0–1882) than transvenous technique (334 days, range 0–2745) is most likely due to the inclusion of post-operative deaths due to euthanasia, which are not necessarily technique related. The cumulative survival with the event defined as death related to pacemaker complication included 9 dogs when all other deaths (n = 26) were censored. This makes the analysis vulnerable to type I error. In this study, dogs with epicardial PPI had a significantly shorter cumulative survival as compared to dogs with transvenous PPI, however, when only the first pacemaker survival was analyzed, there was no difference in cumulative survival probability between techniques (P = 0.07). This likely highlights that apparent lower survival probability for epicardial PPI technique was likely biased, and the negative association with outcome is in part related to it being considered in this institution a salvage technique in those cases with acute loss of capture in the absence of dislodgement. A previous study’s conclusion is in agreement with our results, with no difference in complication rate and cumulative survival between epicardial and transvenous implantation technique [16]. In our study, of the pacemaker related deaths, 55% (5/9) had an epicardial lead and 33% (3/9) had a transvenous epicardial lead in place at the time of death. The differences in epicardial and transvenous pacemaker related deaths were influenced by numerous anomalous circumstances. The specific technique for PPI was not the cause of pacemaker failure in three cases, and in one case the technique associated with death was assigned erroneously.

The effect of age, sex, indication for pacing, and presence of concurrent heart disease on cumulative survival were not significant (age P = 0.4061, sex P = 0.9418, structural heart disease P = 0.2593, indication for pacing P = 0.3121). This is in contrast to previous published results which demonstrated that older dogs, dogs over 10 kg in the case of epicardial PPI, and the presence of congestive heart failure have a shorter survival [7, 15, 16, 36]. One report found middle-age dogs survived longer than old and young dogs [20].

The effect of a major complication on survival probability (P<0.001) has similarly been noted previously [15]. This mortality rate is similar to a previous report where pacemaker related deaths were 20% of all deaths [16]. Incidence of pacemaker related deaths were lower in previous reports of single institutional studies varying from 4.2% (2/42) [21] to 7% (4/60) [20] of all deaths [20, 22]. The reasons for lower mortality rate related to PPI for these studies are likely related to overall lower complication rate. A trend toward association of mortality with complication rate has been described [15]. The reason for low complication rate recorded by Wess *et al*. (2006) were ascribed to the center being a high-volume implantation center and surgical experience being the likely reason [20]. This could explain the differences noted in our study and that of Oyama *et al*. (2001), in that our center and some contributing institutions would be considered to have a low volume implantation case load and likely experience with the technique is inferior [16]. Major complication rate is lower (14%) in experienced operators [16]. Two studies reported periprocedural mortality of 6.5% (3/31) [24], and 5.8% (3/57) [35], which was similar to our perioperative mortality of 4.8% (4/82) within 24 hours of surgery [24]. The cause of death assigned to pacemaker related in our population of dogs, included a cardiac tumor and an owner refusal to change the pacemaker battery, and when these are excluded the cause of death being pacemaker related is even lower (n = 7). The previous study, which reported a low major complication and mortality rates, stated that six dogs (6/105; 5.7%) died suddenly between 3 and 55 months after implantation [20]. The large multi-institutional study, with similar complication and mortality rate to our study, had 7 sudden deaths (with a follow up period that ranged from 2 days to 60 months) [16]. One dog in our study had sudden unexpected death 40 days after epicardial implantation. In the human literature, adverse reaction of intrinsic ectopic beats not sensed appropriately is the presumed cause of arrhythmic death, accounting for 25% pacemaker associated mortality [37]. Undeniably this procedure has a high complication rate, but we would be overstating that there is a higher risk with one technique over the other. Acute failure to capture appeared to occur with both transvenous and epicardial, as was observed in two cases and, in both cases, euthanasia occurred after failure in repeated attempts, with epicardial PPI placement being the last technique employed.

### Limitations

There are several limitations that the authors recognize, mostly inherent to the nature of a retrospective study, including several omissions in the medical records leading to many absent data points. The population was small, and the possibility remains that the study’s lack of power could have led to a type I error where, due to chance or unrelated factors, a significant difference between the technique of PPI in cumulative survival was detected. The shorter cumulative survival in epicardial is unlikely to be related to the technique but rather the motivation for this technique. In some of the dogs that had short-term death with epicardial PPI also had previous acute loss of capture without dislodgement with transvenous, and this could indicate an exit block that is unrelated to technique. The last pacemaker implanted would have been epicardial and an unfair association with survival.

There are several additional limitations in this study. Statistical comparison in complication rates between the two groups could not be made due to the few instances recorded. The study suffers from the selection bias inherent in a retrospective study, particularly related to the lack of standardized care and variation in owners’ willingness to treat their pets or variation in perception of quality of life, which creates disparity when humane euthanasia is elected. The direct comparison between epicardial versus transvenous approach may have been biased as the decision could have been influenced by whether this was in office hours or out of hours and staffing availability. The exclusion criteria eliminated only a few dogs from this study (n = 3), all related to lack of clear indication for PPI, as an attempt to avoid potential confounding factors on survival. Previous studies have had variation in reports of complications, some differentiating major from minor and others reporting complications overall. Our study defined a major complication as life-threatening if not corrected. Conditions including lead thrombus, sensing abnormalities and seroma were not considered major complications. The inferences regarding the differences found in the type of temporary pacemaker technique (transthoracic versus transvenous), in terms of complications and effectiveness of pacing, could not be examined. There was no data on the placement of the pacing electrodes and complications for transthoracic was not described further than failure to capture; no description was included detailing attempts to replace the position of the electrodes. In addition, there was no indication why one technique was used over the other i.e., based on body size, thoracic wall shape or the type of PPI technique employed. Our study found equal number of complications between transthoracic and transvenous temporary pacing approaches. A retrospective study examining transvenous versus transthoracic temporary pacing found both techniques to be efficient in temporary pacing, however, transvenous increased the time of the procedure by delaying the duration from pre-medication to anesthesia induction [38]. This data was not collected in this study. Lastly, several clinicians contributed over time to cases that were included in the study. With lack of standardized technique, this variance may have contributed to outcome differences.

## Conclusions

This retrospective descriptive study detailed the complications in both epicardial and transvenous PPI. No significant association was found between the clinical signs and the choice of PPI technique and pacemaker associated death. The cumulative survival with an event being death directly related to the pacemaker found that epicardial was significantly shorter survival than transvenous. This was likely skewed by the inevitable replacement of a transvenous PPI with an epicardial lead, where lead dislodgement was not the cause of acute loss of capture i.e. rising threshold, and the problem persisted. When dogs with only the first PPI were considered, this difference was no longer apparent. Both techniques offer reliable management of artificial pacing in dogs for a variety of bradyarrhythmia. From the results of this and previous studies, it cannot be ignored that PPI is associated with an unacceptable high major complication rate and despite unequivocally having a strong indication for the management of symptomatic bradycardia, these complications should be included as part of the informed consent. Major complications necessitate the need for revision of the procedure, and the associated costs involved are high. Due to similar incidence of complications between the two techniques, the authors feel it is reasonable to advocate for PPI via epicardial technique in facilities that lack personnel with minimally invasive technique expertise or fluoroscopy, or when geographic constraints in decision making play a role.

## Supporting information

S1 File The data recorded for the 66 dogs that underwent permanent pacemaker implantation (PPI) to correct symptomatic bradycardia at a single center detailing the complications. The excel file categorizes the data under the following sheets: signalment and complaint; cardiac pacing, temporary pacing, anesthesia, surgery, and outcome. The cardiac pacing sheet lists the findings per visit rather than per PPI as is the case for the other sheets.
(XLSX)
